# Cutting Edge of Traumatic Maculopathy with Spectral-domain Optical Coherence Tomography – A Review

**Published:** 2015

**Authors:** Sílvia Mendes, António Campos, Joana Campos, Arminda Neves, Diana Beselga, Cristina Fernandes, João Paulo Castro Sousa

**Affiliations:** Ophthalmology Department - Leiria Hospital Centre, Portugal

**Keywords:** Ag Berlin’s Edema, Commotio Retinae, Spectral-domain Optical Coherence Tomography, Traumatic Maculopathy

## Abstract

This article reviews clinically relevant data regarding traumatic maculopathy (TM), frequently observed in clinical practice, especially due to sport or traffic accident injuries. It is characterized by transient gray-whitish retinal coloration and reduction of visual acuity (VA) with closed, blunt object globe trauma of their prior. It may be limited to the posterior pole (Berlin’s edema), or peripheral areas of the retina. Spectral-domain optical coherence tomography (SD-OCT) provides detail insight using high resolution cross-sectional tomographs of the ocular tissue. It is a potent non-invasive tool for the clinician to follow-up. Clinicians are, thereby empowered with a tool that enables evaluation of the retinal status and allows for prediction of the prognosis. Spectral-domain optical coherence tomography supports the idea that the major site of injury is in the photoreceptor and layers of the retinal pigment epithelium (RPE). Depending on the severity of the trauma, SD-OCT may reveal differential optical densities of intraretinal spaces ranging from disappearance of the thin hyporeflective optical space in mild lesions, or areas of disruption of the inner segment/outer segment (IS/OS) junction and hyperreflectivity of the overlying retina, pigment disorders and retinal atrophy, in more severe cases. The prognosis for recovery of vision is generally good, and improvement occurs within 3-4 weeks.

## INTRODUCTION

Commotio retinae (CR) is a condition frequently observed in clinical practice, particularly following closed globe trauma (CGT) due to sport or traffic accident injuries (1-4). It is the main cause of unilateral vision loss in male patients aged between 22-44 (5-7). It is characterized by transient gray-white retinal coloration and reduction of visual acuity (VA) which usually lasts for 3 to 4 weeks (4).

Symptoms depend mainly on the location of the injury, with fewer complains when only the peripheral retina is affected. It may be confined to the posterior pole, when it is also called Berlin’s edema, after the first hypothesis of Berlin (1873) that the findings of the fundoscopic exanimation were due to the extracellular retinal edema (9). In the other hand, it may involve areas of the peripheral retina (1,2). Depending on the severity of the trauma, spectral-domain optical coherence tomography (SD-OCT) can reveal different optical densities of intraretinal spaces. These may range from mild lesions with transient hyper-reflectivity of the outer retina and disappearance of the thin hypo-reflective optical space; to more severe cases with areas of disruption in the inner segment/ outer segment (IS/OS) junction and hyper-reflectivity of the overlying retina, pigment disorders and retinal atrophy (2,3). The prognosis for recovery of vision is good, with improvement within 3-4 weeks (4), but additional pathology may hold the recapture. Other patients may have good VA recovery, but a scotoma may persist (10,11). On the other side, patients may have a complete VA and SD-OCT recovery, but altered macular function for six months after the trauma (12). Spectral-domain optical coherence provides cross-sectional high-depth resolution tomographs (10 µm) of the ocular tissue. It is a potent non-invasive tool for the clinician to follow-ups by means of which they may evaluate the retinal status and establish prognosis. Currently, there is no treatment available for CR.

## PATIENTS AND METHODS

Two authors (SM, JC) independently reviewed all human studies and identified potentially relevant ones published in the Ovid database (1975 – July 2014) with no specific limits (e.g. language, type of papers, etc.). Both retrospective and prospective studies as well as relevant case reports were included. Literature was searched using terms: commotio retinae, Berlin´s edema and traumatic maculopathy, including related terms. Furthermore, reference lists of retrieved articles and review articles were reviewed manually to identify missing relevant publications. Eleven studies were found suitable for inclusion in this review. The characteristics of 11 included studies are outlined in Table 1. Study sizes ranged from 1 to 170 patients.

*Characteristics of Included Studies*

The characteristics of 11 included studies are outlined in Table 1. Study sizes ranged from 1 to 170 patients.

## RESULTS

Among those 11 studies, Blach et al. (1) observed hyphema in 24 out of 170 patients that suffered closed globe trauma, three of those patients were reported to have retinal tears, as well. In the study of Ahn et al. (3) microhyphema or traumatic iritis was identified in 40 out of 49 eyes (81,6%). Two patients have had extrafoveal choroidal rupture, three extrafoveal subretinal hemorrhage and two mild vitreous hemorrhage. Useful thing is the four-grade grading system (Table 2) they had developed that is used widely to document the baseline severity of photoreceptor damage and predicting visual and anatomic outcomes. Souza-Santos et al. (2) reported intraretinal hemorrhages in 60% of the cases with persistent visual impairment. Saleh et al. (12) showed that CR is associated with an increase in reflectivity of the IS/ OS photoreceptor junction in OCT due to horizontalization of OS in CR. The deformation of the globe during a high-speed impact has been extensively studied and the greatest shearing forces resulting from shock waves appear by the posterior vitreous base (14). In that same study, the OCT revealed a normal central macular thickness in all cases of CR presentation. Souza-Santos et al. (2) demonstrated that mild lesions showed transient hyperreflectivity of the outer retina implicating good prognosis, while disruption of the IS/ OS junction in more severe trauma, especially with hyper-reflectivity of the overlying retina, pigment disturbance, retinal atrophies were associated with poor vision prognosis. Among a more up-to-date molecular basis for retinal changes in CR, macrophages had been under scrutiny for their several functions and various phenotypes (15). 

**Table 1:** Characteristics of the Eleven Included Studies

**Author**	**Year**	**Type of study**	**N (patients)**	**Median age**	**Initial VA**
**Blanch RJ, et al.**	2013	Retrospective Case series	170	28 * 23**	20/40 20/30
**Saleh M, et al.**	2011	Retrospective Case series	20	20.8	20/100
**Seong JA, et al.**	2013	Retrospective Case series	49	32	N/A
**Souza-Santos, et al.**	2012	Prospective Observational	11	30.8	CF – 20/20
**Thach, et al.**	1999	Retrospective Case series	13	21	LP – 20/50
**Khan, et al.**	2011	Retrospective Case series	10 (11 eyes)	11 (Pediatric study)	LP – 20/20
**Pham TQ, et al.**	2007	Case reports	2	Patient 1 - 30 years old Patient 2 - 43 years old	Patient 1 – 20/200 Patient 2 – 20/120
**Oladiwura D, et al.**	2014	Case report	1	27	20/15
**Mendes S, et al.**	2014	Case report	1	12	CF
**Park JY, et al.**	2011	Retrospective Case series	9	22,5	20/40 – 20/20
**Noia L, et al.**	2006	Prospective	16	23	CF – 20/16

CF – counting fingers at 1 m

LP – Light perception

* Macular commotio retinae

** Extramacular commotio retinae

In the vein of Berlin’s hypothesis (9) that was later found over-speculative (16,17), more up-to-date molecular bases for retinal changes were kept looking for. Sipperley et al. (17), for instance, reported active phagocytosis of the fragmented OS by RPE 24 hours after the trauma, formation of multiple layers of RPE cells on Bruch’s membrane and migration into the neurosensory retina. According to the review of Viestenz et al. (21), 20% of CGTwas associated with retinal detachment, 57% with vitreous hemorrhage, 86% with hyphema, 43% with iris or cyclodialysis, 21% with intra or subretinal hemorrhage and traumatic cataract in 41%. They also found a strong positive correlation between previous cataract surgery and globe rupture after CGT(27 fold).

Histologic analysis showed that the major site of injury in CR involves photoreceptor OS and RPE layers (16,17,22). On the other hand, several studies showed reduced amplitudes of ERG in traumatic maculopathy (12,23,24). Noia et al. (19) described more specifically the ERG profile of 16 CR patients. The study offered statistical significant difference in responses between eyes. 

**Table 2:** Complete Recovery of all the Three Photoreceptor Layers, in Ahn et al. (3).

**Grade**	**Description**	**Final VA**	Anatomic Restitution*
**1**	Decrease of the reflectivity of IS/ OS junction disappearance of the hypo-reflective OS	All eyes ≥20/20	100%
**2**	Cone outer segment tips (COST) defect only	~50% ≥20/20 ~50% ≤20/200	100%
**3**	COST and IS/OS junction defects	~25% ≥20/20 ~75% ≤20/200	14,3%
**4**	COST, IS/OS junction and external limiting membrane defects	All eyes ≤20/200	5,9%

## DISCUSSION

Commotio retinae is a frequent cause of ophthalmological emergency following CGT, mostly due to sport, but to the traffic or work accidents. There is no specific treatment, but clinicians usually treat anterior and posterior segments-associated injuries. Usually, it brings along a favorable outcome in regard to eyesight, what depends mainly on the extent and location of retinal injury. In recent years gladly performed SD-OCT (2,3) supports the findings of the histologic examinations, identifying photoreceptor and RPE layers to be the major site of injury (25). Sometimes, a cherry-red spot may appear in the posterior retina because the cells involved in CR are not present in the foveola (4). Spectral domain optical coherence tomography can reveal transient hyper-reflectivity of the outer retina and disappearance of the thin hypo-reflective optical space in mild lesions, or areas of disruption of the inner/outer segment (IS/OS) junction and hyperreflectivity of the overlying retina, pigment disorders and retinal atrophy in more severe cases. It is a potent non-invasive tool that provides high-depth resolution cross-sectional tomographs, and it represents a substantial progress in resolution and speed of image acquisition, allowing for the evaluation of photoreceptors on a microscopic level, allowing the evaluation of the retinal status, prognosis and follow-up.

*a) Pathogenesis*

The pathogenesis of CR has been proposed to be both mechanical and hemodynamic (13). The deformation of the globe during a high-speed impact has been extensively studied, and greatest shearing forces caused by shock waves were demonstrated in the retina are along the posterior vitreous base (14). Often, macular CR may be associated with orbital floor fractures, probably due to retropulsion and increased intraorbital pressure, while extra-macular CR (predominately in the temporal area) is more associated with orbital rim fractures, consistent with direct trauma to the overlying sclera (1,15). On the other hand, it is possible that hydraulic forces transmitted posteriorly, associated with vitreous deformation, would cause different movements between different density tissues and would be responsible for additional damage. The tissues more prone to suffer from these differences are the vitreous-retina and photoreceptor-RPE interfaces. Exactly in this vein could be the discoloration of the retina interpreted (12), what is followed by the irregular pieces of photoreceptor material, which replaces the well-aligned outer segments (18).

As an additional moment in the pathogenesis of CR would be the contribution of Sipperley et al. (17), who reported active phagocytosis of the fragmented OS 24 hours after the trauma. Intraretinal hyper-reflexive aggregates visible in SD-OCT of severely affected areas may represent clusters of these RPE cells. Later, total loss of the OS photoreceptors was observed in those areas, what was accompanied by thinning of the outer plexiform and outer nuclear layers. 

*b) Associated Injuries*

Closed globe trauma causes direct anterior segment injuries such as corneal injury, iris sphincter tears, angle recession, cyclodialysis or trabecular meshwork lesions. Additionally, it is associated with a great number of retinal complications in the posterior segment as retinal detachments or dialysis, vitreous hemorrhage, choroidal ruptures, traumatic macular holes or traumatic optic neuropathy. These associated injuries in CR may limit VA and anatomical integrity restitution. In the majority of the studies, microhyphema (blodd cell Tyndall) or hyphema and corneal erosions were the injures most frequently associated with CR (1,12,19,20).

Ahn et al. (3), Souza-Santos et al. (2), Blach et al. (1) all reported findings corroborating the review of Viestenz et al. (21) it was referred that 20% of CGT in the Erlanger Okuläres Contusions-Register was associated with retinal detachment, 57% with vitreous hemorrhage, 86% with hyphema, 43% with iris or cyclodialysis, 21% with intra or subretinal hemorrhage and traumatic cataract in 41%. They also found a strong positive correlation between previous cataract surgery and globe rupture after CGT (27 fold).

*c) Optical Coherence Tomography (OCT)*

Histologic analysis showed that the major site of injury in CR involves photoreceptor OS and RPE layers (16, 17, 22) Optical coherence tomography (OCT) is a powerful non-invasive tool for evaluating the retinal status. Compared with the less complex, an optical signal acquisition and processing method called time-domain OCT, SD-OCT provides cross-sectional tomographs of high-depth resolution (10 µm) of the ocular tissue. What is more, SD-OCT brings a substantial progress in resolution and speed of image acquisition allowing for the evaluation of the photoreceptors on a microscopic level.

In the wake of more sophisticated optical signal acquisition and processing methods emerging daily, Saleh et al. (12) showed that CR is associated with an increase in reflectivity of the IS/ OS photoreceptor junction visible on OCT with disappearance of the hypo-reflective optical space. Souza-Santos et al. (2) demonstrated that mild lesions showed transient hyper-reflectivity of the outer retina, finding that they associated with good prognosis. More severe trauma was, in contrast, accompanied by the disruption of the IS/ OS junction and findings like hyper-reflectivity of the overlying retina, pigment disturbance, retinal atrophy, what corresponds to poor sightedness prognosis. 

Even more recently, Ahn et al. (3) investigated the morphologic changes in SD-OCT of 49 patients with CR and developed a grading system for documenting the baseline severity of photoreceptor damage and predicting vison and anatomic integrity outcomes with 4 step grading system. Higher grades at baseline were associated with worse final visual and anatomic outcomes (See Table 2).

*d) Electroretinogram (ERG) Findings*

Several studies showed reduced amplitudes of electroretinogram (ERG) in traumatic maculopathy. (12, 23, 24) Noia et al. (19) described more specifically the ERG profile of 16 patients who had suffered CR in the past 72 hours. The study presented a decrease in wave amplitude in all the 5 steps of ERG (rod response, maximal combined response, oscillatory potentials, single-flash cone response and 30-Hz flicker response). There was a statistical significant delay in single-flash cone response and 30-Hz flicker response in the eye injured compared with the fellow eye. The b wave to a wave relationship may be a useful index for evaluating the ERG. A plot of the b wave amplitude as a function of the a wave amplitude describes the functional integrity of the retina. The b/ a relation was similar between the traumatized and the “intact” eye. The reduction in amplitude was more evident in rod response (less 47% than in the “intact” eye). One month after injury, no significant statistical differences were found between the 5 steps of ERG.

*e) Fluorescein Angiogram and Fundus Autofluorescence*

In the area of CR fluorescein angiography typically shows no evidence of alterations in retinal vascular or choroidal permeability. Hyper-fluorescence areas due to window defect or hypo-fluorescence areas secondary to a blocking effect can, however, be recorded. (19) After reversion of the outer retinal whitening, angiography may or may not show evidence of window defects in the RPE. Mild injuries may show hyper-autofluorescent dots on fundus auto-fluorescence, corresponding to irregularities of the outer layers on SD-OCT. Areas of pigment anomalies may reveal reduced or increased auto fluorescence (2).

*f) Vision Outcomes*

At the time of presentation, VA can range from light perception to 20/20 (1, 3, 12, 19, 20, 25-28), what usually improves quickly. Good vision outcomes depend mainly on the extension and location of retinal injury. Patient is, naturally, complaining less if only the peripheral retina is affected. The grading system developed by Ahn et al. (3) for documenting the baseline severity of photoreceptor damage and predict visual prognosis suggested that the recovery of all layers was more probable in eyes with intact photoreceptor inner segments (grade 1 and 2), and there is a strong correlation between COST defect size and both baseline and final VA. A scotoma may persist up to six months after the trauma (10, 11) or a complete VA and SD-OCT findings recovery may occur in spite of altered macular function (12). 

Blanch et al. (1) described the prognosis and retinal location in patients with acute traumatic maculopathy (53 patients) and extramacular retinal injuries (117 patients). In the macular CR group, mean VA at presentation was 20/40, 74% recovered to ≥ 20/30 and 24% remained <20/30. Patients with final VA <20/30 had concomitant macular hole (1 patient), macular choroidal folds secondary to hypotony (1 patient) or outer retinal atrophy. In the extramacular retinal CR group median VA at presentation was 20/30 with mean final VA of 20/20. Three patients had final VA <20/30. Of these patients, 1 had a past history of amblyopia with no change in vision from the preinjury VA and another had an extramacular choroidal rupture. Associated injuries were hyphema (12 patients), retinal tears (3 patients), retinal dyalisis (1 patient) and orbital fractures (13 patients).

*g) Long term Outcomes*

Several publications of case reports described short-term findings of SD-OCT and clinical examination in CR, but only one prospective study described the long term consequences (6 month-long follow-up) on macular function in 20 patients (12). In this study the mean central thickness was not statistically significant from baseline (161.9 µm vs 155.9 µm). The macular alterations were restored at 6 months in most patients, but in 3 cases mild disruptions in the photoreceptor layer persisted. In the same study, it was found multifocal electroretinogram defects (decrease in central wavelet amplitude with a relative preservation of the implicit times) despite restored VA. One article 29 reported a case of a 12-year-old male 6 months after injury presenting a fundus with a central macular lesion with 1 disk diameter, fibrosis, increased retinal thickness and intraretinal hemorrhages. SD-OCT showed disruption of the IS/OS junction with corresponding increased reflectivity, loss of the outer nuclear layer, cell infiltration of the retinal wall and subretinal fibrosis. Fluorescein angiogram revealed early impregnation with no diffusion increasing with time. After treatment with high dose steroid pulse therapy and intravitreal triamcinolone (IVTA) (0.05 mL/2 mg) retinal thickness decreased and VA improved, although fibrosis and disruption of the IS/OS junction persisted. Others studies reported damage of the photoreceptor layer and the RPE several months after CR, both in animal models and in humans (17, 30)

## CONCLUSION

Vision and anatomic integrity recoveries are favorable in most cases of CR. Associated choroidal neovascularization, traumatic optic neuropathy or macular hole in macular lesions may limit recovery, as retinal detachment or ruptures in periphery. Considering the average age of the patients with CR and a long life expectancy, the prognosis and long-term complications need to be further evaluated. And techniques using OCT technology are certainly pivotal to that evaluation. 

## DISCLOSURE

The authors report no conflicts of interest in this work.
